# Cerebral Microvascular Pathology Is a Common Endophenotype Between Traumatic Brain Injury, Cardiovascular Disease, and Dementia: A Hypothesis and Review

**DOI:** 10.7759/cureus.25318

**Published:** 2022-05-25

**Authors:** Randel L Swanson, Nimish K Acharya, David X Cifu

**Affiliations:** 1 Center for Neurotrauma, Neurodegeneration and Restoration, Corporal Michael J. Crescenz VA Medical Center, Philadelphia, USA; 2 Physical Medicine and Rehabilitation, Perelman School of Medicine, University of Pennsylvania, Philadelphia, USA; 3 Cell Biology and Neuroscience, Geriatrics and Gerontology, New Jersey Institute for Successful Aging, Rowan University School of Osteopathic Medicine, Stratford, USA; 4 Physical Medicine and Rehabilitation, Virginia Commonwealth University School of Medicine, Richmond, USA; 5 Physical Medicine and Rehabilitation, Central Virginia Veterans Health Affairs System, Richmond, USA

**Keywords:** dementia, cardiovascular disease, blood-brain barrier, neurovascular unit, cerebral microvascular pathology, traumatic brain injury

## Abstract

Traumatic brain injury (TBI) exposure has been associated with an increased risk of age-related cognitive decline or dementia in multiple epidemiological studies. Current therapeutic interventions in the field of Brain Injury Medicine focus largely on episodic symptom management during the chronic phase of TBI recovery, rather than targeting specific underlying pathological processes. This approach may be especially problematic for secondary age-related cognitive decline or dementia following TBI exposure. Although there are likely multiple pathophysiological mechanisms involved, a growing body of literature demonstrates that cerebral microvascular pathology is a common endophenotype across the spectrum of TBI severity. Similarly, a combination of pre-clinical and clinical research over the past two decades has implicated cerebral microvascular pathology in the initiation and progression of multiple neurodegenerative diseases, including Alzheimer’s disease and other dementias. We hypothesize that cerebral microvascular pathology is a common endophenotype between TBI, cardiovascular disease (CVD), and dementia, which can be targeted through modifiable cardiovascular risk factor reductions during the chronic phase of TBI recovery. We posit that our hypothesis is supported by the currently available scientific literature, as detailed in our review.

## Introduction and background

More than 2.5 million new cases of traumatic brain injury (TBI) are diagnosed annually in the U.S. and an estimated 11 million individuals are living with long-term TBI-associated disability [1,2]. Epidemiological studies have demonstrated an association between TBI exposure(s) and an increased risk of age-related neurocognitive decline or dementia [3-7], including a 2018 retrospective cohort study involving over 350,000 military veterans which found more than a two-fold increased risk of developing dementia amongst individuals with mild TBI exposure and more than a 3.5-fold increased risk of developing dementia amongst individuals with moderate-to-severe TBI exposure [6]. However, the pathophysiological mechanism(s) that underlies the increased dementia risk in TBI survivors remains unclear. Given the magnitude of this problem, there is a significant need to identify modifiable risk factors that contribute to secondary neurocognitive decline with aging following TBI exposure(s).

A 2020 report from the Lancet Commission on Dementia Prevention, Intervention, and Care summarized the growing body of scientific evidence related to potentially modifiable risk factors in the development of dementia [8]. This report identified multiple cardiovascular factors, such as diabetes, hyperlipidemia, hypertension, obesity, smoking, excess alcohol consumption, poor diet, and infrequent physical activity, among others, which are known to modulate the initiation and progression of atherosclerotic cardiovascular disease (i.e., heart disease and stroke) [9-12]. In a large, retrospective cohort study of U.S. Military Veterans [13], Barnes et al. demonstrated that while the cumulative incidence of dementia diagnosis as a function of age was higher among Veterans with isolated TBI or isolated cerebral vascular disease (i.e., a manifestation of broader underlying cardiovascular disease (CVD)) than in Veterans without either singular diagnosis, the dementia incidence was significantly greater among Veterans with the dual-diagnoses of TBI plus comorbid CVD than with either independent diagnosis [13]. Further, mounting evidence suggests that modifying cardiovascular risk factors through either lifestyle or pharmacological interventions, particularly in midlife, could significantly lower the risk of developing age-related cognitive decline and dementia [8,14] by up to 60% for individuals with the healthiest cardiovascular risk factor profiles [14].

Hypothesis

**Figure 1:**
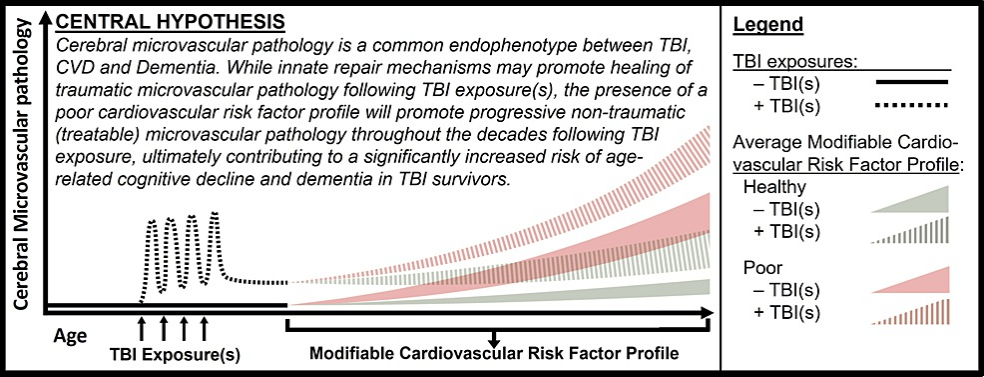
Schematic Representation of Our Central Hypothesis TBI, Traumatic Brain Injury; -TBI(s), no TBI exposure(s); +TBI(s), positive TBI exposure(s); CVD, Cardiovascular Disease.

We posit the following *Central Hypothesis*: Cerebral microvascular pathology is a common endophenotype between TBI, CVD, and Dementia. While innate repair mechanisms may promote healing of traumatic cerebral microvascular pathology following TBI exposure(s), the presence of a poor cardiovascular risk factor profile will promote progressive, non-traumatic, cerebral microvascular pathology throughout the decades following TBI exposure, ultimately contributing to a significantly increased risk of age-related cognitive decline and dementia in TBI survivors (Figure 1).

## Review

The following review aims to evaluate our stated hypothesis, based on the currently available scientific literature.

The neurovascular unit

The cerebral microvasculature is unique compared to the microvasculature of any other organ, in that it is comprised of an interdependent combination of vascular and neuronal components, collectively referred to as the Neurovascular Unit (NVU). First defined in 2001 [15], it is now established that the NVU plays a critical role in maintaining homeostasis within the brain and is a key element in the pathophysiology of numerous neurological disorders, including Alzheimer’s disease (AD) and other dementias [15-19]. Vascular components of the NVU include endothelial cells, pericytes, and a basement membrane at the capillary level, with pericytes replaced by smooth muscle cells at the arteriole, venule, and artery levels. Throughout all cerebral microvascular “levels,” the endothelial cells form a continuous monolayer on the innermost aspect of the vessel, which is interconnected with either tight junctions or adherens junctions to form the blood-brain barrier (BBB). Neuronal components of the NVU include perivascular astrocytes, along with perivascular nerves terminating on or near astrocytic foot processes for capillaries, or on smooth muscle cells for arterioles and arteries. Collectively, a key function of the NVU is to rapidly couple the metabolic demands of various brain regions with the appropriate amount of blood supply, thereby regulating regional cerebral blood flow and modulating cerebrovascular reactivity. NVU dysfunction can: 1) result from pathology within any of the neuronal or vascular components; 2) occur acutely as with TBI exposure or be slowly progressive as with CVD; and 3) be mechanical or functional in nature [15-18]. A common consequence of both traumatic and non-traumatic insults to the NVU is disruption of the BBB. Perturbed BBB function results in the extravasation of blood products into the brain parenchyma. Consequently, chronic extravasation of vascular components both initiate and perpetuate detrimental downstream neuropathological consequences [17,18].

TBI and cerebral microvascular pathology

A growing body of evidence suggests that compromise to the NVU can be a primary (mechanical) and/or secondary (functional) consequence of TBI, across the spectrum of TBI severity [17,20-28]. Pre-clinical TBI models encompass a wide variety of injury mechanisms and severities, all of which have documented TBI-induced cerebral microvascular pathology [24,25,29,30]. Among lissencephalic rodent models, TBI exposure with weight drop, controlled cortical impact, fluid percussion injury, and blast overpressure models have all documented cerebral microvascular pathology at the level of the NVU and BBB [24,29]. Further, more relevant to human TBI pathophysiology, large animal swine models have been used in pre-clinical TBI research due to their gyrencephalic brain architecture, large brain mass, higher ratio of white to gray matter, and the ability to undergo head rotational acceleration injury similar to most forms of human TBI exposure [30]. Even on the mild end of the TBI spectrum, acute mechanical disruption of the NVU following single, non-impact, rotational acceleration injury (RAI) TBI in swine has been demonstrated [25]. Specifically, BBB compromise was revealed histologically by the presence of extravasated blood products (i.e., Fibrinogen and Immunoglobulin-G) from the cerebral microvasculature into the brain parenchyma acutely after a single RAI mild-TBI, in a pattern consistent with the direction of the biomechanical injury [25].

Human studies of TBI-induced cerebral microvascular pathology include both structural and functional NVU assessments [26-28]. In human autopsy studies using the Glasgow TBI Archive, Hay et al. performed a neuropathological evaluation of NVU structural integrity (based on analysis of fibrinogen immunoreactivity) in 70 cases of moderate-to-severe TBI exposure and documented focal mechanical NVU/BBB compromise in 88% of acute survival cases and 69% of long-term survival cases, as compared to only 19% in age-matched uninjured controls [26]. Evaluating the long-term functional consequences of traumatic NVU pathology, another group of scientists began investigating cerebrovascular reactivity using a combination of functional magnetic resonance imaging (fMRI) and functional Near InfraRed Spectroscopy imaging [27,28]. Preliminary results among 21 subjects more than six months after a moderate-to-severe TBI exposure demonstrated a significant functional decrease in cerebrovascular reactivity compared to matched healthy controls [28]. Considering these human and animal studies collectively, NVU pathology likely contributes to chronic TBI-related sequelae and may underly the observed increased incidence of dementia among TBI survivors [3-7].

CVD, cerebral microvascular pathology, and dementia

Over the past 25 years, there has been an exponential increase in our understanding of the pathophysiological role CVD and cerebral microvascular pathology play in the initiation and progression of multiple neurodegenerative diseases [18]. With numerous pre-clinical and clinical dementia studies investigating blood-based biomarkers, advanced neuroimaging, and neuropathological markers of cerebral microvascular injury, NVU dysfunction has been widely implicated in a variety of dementia subtypes, including AD [19,31-34], frontotemporal dementia (FTD) [19,35-38], and dementia with Lewy Bodies (DLB) [39-41]. Evidence from blood-based biomarkers studied include a 2017 Swedish-based study that utilized the clinical gold-standard cerebrospinal fluid/plasma albumin ratio (Qalb) to evaluate the extent of NVU pathology among patients with a clinical dementia diagnosis [19]. This study demonstrated a significant increase of Qalb in patients diagnosed with AD, FTD, DLB and vascular dementia, indicating increased BBB permeability compared to controls [19]. More recently, studies utilizing dynamic contrast-enhanced MRI to evaluate regional NVU permeability have documented age-related NVU dysfunction in the hippocampus, which is further increased in patients with mild-cognitive impairment [32,42].

While a multitude of studies have implicated CVD and modifiable cardiovascular risk factors in the pathogenesis of AD and other dementias, well-controlled large-scale neuropathological studies were lacking until a collaborative group of neuropathologists, in 2013, systematically evaluated autopsy specimens from the National Alzheimer’s Coordinating Centre database, including 4,629 cases with confirmed Alzheimer’s disease [34]. The study documented the presence of cerebrovascular pathology in 79.9% of cases of pathologically confirmed AD [34]. Further, the study divided cases by age and demonstrated an age-dependent increase in the prevalence of cerebrovascular pathology [34]. Taken together, there is a substantial body of research documenting the relationship between CVD, cerebral microvascular pathology, and dementia.

Modifiable cardiovascular risk factors and resulting microvascular pathology

In 2010, the American Heart Association (AHA) formed a task force to develop the concept of “cardiovascular health,” and to determine the evidence-based modifiable metrics that impact CVD [11]. Their conclusions led to the development of the AHA’s Ideal cardiovascular health (I-CVH) index, which was refined slightly in 2020 [12]. Four *Ideal Health Behaviors *(physical activity, body mass index, smoking, and diet) and three* Ideal Health Factors* (blood pressure, total cholesterol, and diabetes status) are each categorized into one of three pre-defined groups, indicating an Ideal (2 points), Intermediate (1 point) or Poor (0 points) cardiovascular metric for the given behavior/factor [12]. The sum of these seven metrics generates a single ideal-CVH index ranging from 0-14, with a score of 14 indicating Ideal Cardiovascular Health [12]. A subsequent study performed on a large cohort of healthy adults (n = 65,949, mean age 41.3, 78.7% male) followed for over five years found an inverse association between I-CVH Index, and the presence and five-year progression of subclinical atherosclerotic vascular disease, as measured by coronary artery calcium imaging [9]. Further, over the past decade, there have been several studies (both pre-clinical and clinical) documenting the relationship between modifiable cardiovascular risk factors and the development of cerebral microvascular pathology, or the downstream sequelae of clinically diagnosed dementia [10,14,43-49].

Pre-clinical studies investigating the combined impact of Diabetes and Hypercholesterolemia in a clinically relevant swine model provide direct evidence that the presence of these two modifiable cardiovascular risk factors produces both peripheral and cerebral microvascular pathology [45-48]. Specifically, the diabetic and hypercholesterolemic swine demonstrated both BBB pathology [45] and blood-retinal barrier (BRB) pathology [46] compared to the age-matched non-diabetic and -hypercholesterolemic control swine.

A prospective cohort study conducted among 17,761 individuals (aged 45+ with normal baseline cognitive status measured via tests of verbal learning, memory, and verbal fluency) assessed participants at baseline and an average of four years later for both AHA I-CVH Index and incident cognitive impairment [10]. The study documented a significantly lower odds of developing objective cognitive impairment among individuals with intermediate or high scores on the AHA I-CVH Index [10]. A separate study conducted in a baseline stroke-free / dementia-free subset of the Framingham Heart Study Offspring cohort (n=2750, mean age 62.9, 45% male) looked at the 10-year risk of incident stroke and dementia and found remote (> 7-years prior) adherence to the AHA I-CVH guidelines was associated with a subsequent decreased incidence of Alzheimer’s disease and other dementias [49]. Thus, there is a mounting body of scientific evidence demonstrating associations between modifiable cardiovascular risk factors, cerebral microvascular pathology, and cognitive impairment or dementia.

## Conclusions

Research directly evaluating the relative contributions of TBI exposure(s) (i.e., fixed risk factor) and modifiable, co-morbid CVD or cardiovascular risk factors in the development of chronic cerebral microvascular pathology could profoundly impact the clinical practice of Brain Injury Medicine (BIM). While some BIM practitioners are beginning to implement CVD risk reduction in the routine management of chronic TBI survivors, direct evidence of therapeutic / outcome benefit from well-controlled pre-clinical and clinical research studies is needed to move the field toward the routine implementation of aggressive modifiable cardiovascular risk factor reduction among TBI survivors, in an effort to minimize secondary age-related cognitive decline and/or dementia risk. It is the goal of this manuscript to encourage such research, both in the pre-clinical and clinical research settings.
